# The structural relationship between achievement goal orientation and perceived performance among swimmers: a chain multiple mediation model

**DOI:** 10.3389/fpsyg.2025.1536743

**Published:** 2025-05-06

**Authors:** Yuanjiao Zhu, Weipeng Zhang, Hao Liu, Huitao Song

**Affiliations:** ^1^School of Physical Education and Health, Tianjin University of Traditional Chinese Medicine, Tianjin, China; ^2^School of Sports Sciences, Tianjin Normal University, Tianjin, China; ^3^Physical Education, Huanggang Normal University, Hubei, China

**Keywords:** competitive swimming, goal orientation, motivation in sports, sports psychology, structural equation modeling, achievement goal theory

## Abstract

**Background:**

Achievement goal orientation (AGO) has been identified as an important determinant of Perceived Performance (PP) in athletes. However, there is limited research analyzing this relationship through multiple mediation models.

**Methods:**

In this study, data from 377 competitive swimmers (45.1% female, 54.9% male, aged 18–22 years old) from Tianjin and Beijing universities were collected through questionnaires, and structural equation modelling (SEM) was used to analyze the relationship between AGO, Sports Enthusiasm (SE), Sports Commitment (SC), and Grit and PP.

**Results:**

AGO significantly elevated PP (*β* = 0.116, *p* ≤ 0.001), explaining 76.6% of the total effect of AGO on PP through the chain-mediated effects of SE (*β* = 0.472, *p* ≤ 0.001), SC (*β* = 0.448, *p* ≤ 0.001) and Grit (*β* = 0.165, *p* ≤ 0.001).

**Conclusion:**

The chain mediation model of this study suggests that AGO enhances swimmers ‘PP through SE, SC, and Grit, guiding coaches’ goal setting, swimmers ‘progress tracking, and psychologists’ reassessment strategies.

## 1 Introduction

Swimming receives considerable attention in the Olympic Games due to its rich history and demands on athleticism, ranking among the most prominent sports alongside track and field (Lohn, 2021). As competitive standards in swimming intensify, psychological factors increasingly influence performance outcomes at elite levels. In swimming, PP, as a central component of psychological factors, directly affects the performance and mental state of the athlete. Research indicates that swimmers’ PP (appraisal of their abilities) influences stress adaptation and mental toughness (De Meester et al., 2020). As a key indicator of a swimmer’s mental state, PP not only influences its outcome but is also driven by specific psychological factors. AGO enhances PP by setting success criteria, stimulating intrinsic motivation, enhancing stress resilience and self-confidence (Zourbanos et al., 2014; D’Astous et al., 2020), and viewing difficulties as growth opportunities (Lin et al., 2021). In addition, Goal Setting Theory (GST) suggests that clear and challenging goals reinforce SC by increasing focus and effort (Chevance et al., 2021; Jeong et al., 2023). SC maintains a high level of engagement and promotes PP by bringing confidence and a sense of achievement upon task completion (Karlen et al., 2019; Raimundi et al., 2024).Therefore, we propose:

*Hypothesis 1:* AGO positively influences PP.

*Hypothesis 2:* SC mediates the relationship between AGO and PP.

To further investigate how AGO influences PP, SE was introduced as a mediating variable based on a review of related literature. Emotion Regulation Theory (ERT) suggests that positive emotions enhance an individual’s ability to adapt and cope with challenges by regulating their psychological state (Gross, 2015). SE is a high-intensity positive emotion characterized by strong emotional commitment and sustained motivation towards physical activity (Lavoie et al., 2021). This emotional state helps athletes stay focused on performance and manage stress and uncertainty during training and competition (Schellenberg et al., 2024). This aligns with the study’s emphasis on positive motivation, which underpins AGO - the sustained pursuit of success through setting standards and charting a path (Zhang et al., 2023; Chen et al., 2024). In contrast, negative motivation (e.g., avoiding failure), alone or in combination with positive motivation, may transiently enhance swimming performance, However, its fleeting and context-dependent nature diverges from the AGO framework (Gabrys and Wontorczyk, 2023). Thus, we prioritize positive motivation over short-term alternatives. Prior research indicates that SE regulates athletes’ psychological states, enhancing motivation and fostering positive self-feedback during competition (Chamorro et al., 2020). This process supports swimmers in achieving their competitive goals through greater concentration and emotional stability, particularly in collegiate sports. Such emotionally driven success strengthens athletes’ sense of mastery and reinforces positive self-evaluations of their abilities (Blijlevens et al., 2018). Furthermore, prior literature suggests that AGO provides individuals with a clear direction for their endeavors, stimulating emotional engagement and sustained motivation for the activity (Roberts and Nerstad, 2020). Athletes exhibit greater SE when they experience growth and success through goal-directed behavior (Lavoie et al., 2021). Based on the above discussion and theories, the following hypotheses are proposed in this study:

*Hypothesis 3*: SE mediates the relationship between AGO and PP.

Grit refers to the sustained effort and persistent determination individuals exhibit to achieve long-term goals (Duckworth et al., 2007). Research indicates that in collegiate swimming, grit enables athletes to remain focused and motivated during intense training and high-pressure competitions, while also enhancing their confidence in their competitive abilities (Moles et al., 2017). Specifically, swimmers with high grit adhere to rigorous training programs and maintain focus under competitive pressure, leading to more positive self-assessments of their performance. In addition, when faced with challenging races or setbacks, they tended to adjust their self-assessment criteria to optimize future swimming performance (Sullivan et al., 2023; Barcza-Renner et al., 2024). This trait allows them to maintain a positive mindset despite adversity and sets them up for long-term success. Additionally, athletes with AGO are more inclined to view effort and learning as the key to success, and this intrinsic motivation drives them to show more sustained effort in the face of challenges (Moore and Weiller-Abels, 2020). Existing studies demonstrate that setting clear goals and engaging in self-regulation enable athletes to sustain effort over extended periods of training and competition, thereby enhancing grit-related performance (Kitsantas et al., 2018; Zimmerman, 2023). Based on this, this study proposes the hypothesis:

*Hypothesis 4*: Grit mediates the relationship between AGO and PP.

SE, SC, and Grit (GRIT) not only serve as mediating variables between AGO and PP but also interact with one another, collectively influencing the relationship between athletes’ AGO and PP. AGO shapes how individuals define success, encouraging them to view exercise as a means of achieving self-improvement and growth (Papaioannou and Krommidas, 2021). This cognitive orientation heightens awareness of the goal-achievement process, enhancing the perceived significance of exercise and fostering positive SE, For example, swimmers with an AGO will gain motivation from perfecting strokes such as the butterfly during practice (Moreno-Murcia et al., 2011; Uzzell et al., 2024). SE is expressed in swimming both as a strong interest in the sport and as a psychological support that helps swimmers stay focused and put in more effort during training and competition (Lopes and Vallerand, 2020). SC drives swimmers to overcome the monotony of repetitive stroke drills, cope with external challenges like cold water temperatures and competitive pressure, and bolster Grit in pursuit of long-term goals, such as Olympic gold medals (Uzzell et al., 2024). It supports swimmers to persevere in boring underwater environments by increasing intrinsic motivation, adapting to high-pressure situations and achieving breakthroughs in prolonged technical and endurance training (Minkels et al., 2023). This Grit is reflected in the way swimmers perceive challenges as opportunities to improve their abilities and further enhance their competitive skills through relentless endeavor, which in turn boosts their confidence in their swimming ability and fosters a more positive assessment of their performance and potential (O’Neil and Hodge, 2020).Therefore, we hypothesize the following:

*Hypothesis 5*: SE, SC, and Grit have chained multiple mediating roles in the relationship between AGO and PP.

Most existing research has focused on the characteristics of PP across various sports, often overlooking a systematic examination of psychological mechanisms unique to swimming as a professional sport (Anderson and Ramos, 2018; Lochbaum et al., 2022), Previous research has typically examined a single psychological variable (e.g., motivation or self-confidence) without sufficient attention to the interlocking effects of multiple factors of AGO, SE, SC, and Grit (Apró et al., 2024). This study aims to investigate the psychological mechanisms underlying PP in swimmers by developing a multiple chain mediation model grounded in achievement goal orientation theory. This approach applies the theory to swimming, systematically analyzing the chained mediating effects of these factors to address the shortcomings of univariate studies. Ultimately, this research seeks to advance theoretical understanding and enhance swimmers’ PP by elucidating the psychological pathways that shape it, thereby offering theoretical support for designing targeted mental training programs.

**Figure 1:**
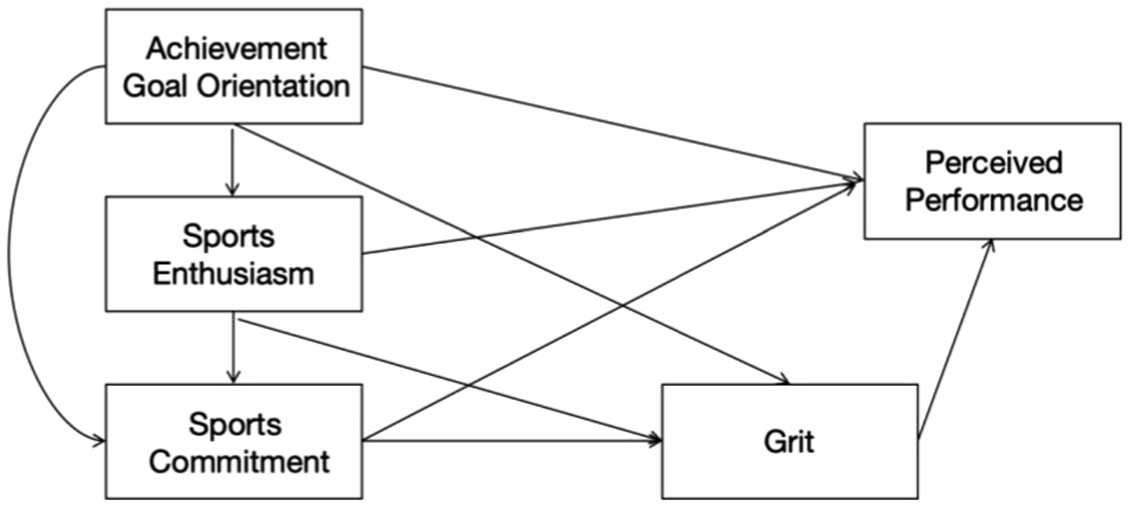
Hypothetical model diagram.

As shown in Figure 1, the hypothesized model demonstrates the specific paths between the variables and their interrelationships, aiming to clarify the structural framework of the research hypothesis.

## 2 Materials and methods

### 2.1 Participants and procedures

This study used a questionnaire to collect data on the variables of Basic Information, AGO, SE, SC, Grit, and PP of college swimmers. The source of the study sample was limited to swimmers from three colleges in Tianjin and three colleges in Beijing. The questionnaire was created by the research team on Questionnaire Star, a professional data collection platform in China, and was recruited through the probability sampling method. During the sample acquisition process, the researcher relied on WeChat, China’s most popular social media platform, to send the questionnaire link to potential participants through the peer network of university sports schools to ensure the coverage and authenticity of the survey. In the end, 377 valid samples were obtained and all participants received a cash prize as a token of appreciation. Of the valid sample, 170 (45.1%) were female and 207 (54.9%) were male; the grade distribution was 159 (42.2%) freshmen, 118 (31.3%) sophomores, 57 (15.1%) juniors, and 43 (11.4%) seniors; and the participants’ ages ranged from 18 to 22 years (*M* = 19.71, SD = 1.25).The distribution of the study population and the characteristics of the sample are representative of the population of collegiate swimmers, which contributes to the external validity of the results. Family factors (e.g., parental support) and personal factors (e.g., personality traits) were excluded from this study, which focused specifically on sport-related psychological predictors within the context of collegiate swimming. Broader social and individual influences were deemed to have limited direct relevance to the study’s primary objectives (Weinberg and Gould, 2023).

All study participants: participated voluntarily and were informed that the survey was anonymous and confidential. Individual consent was obtained from all subjects for this study. All procedures in this study were ethical and in accordance with the 1964 Declaration of Helsinki and subsequent amendments or similar ethical standards. The Ethics Committee of Tianjin Normal University approved this study (2024112501).

### 2.2 Measures

Following Brislin (1986) translation and back-translation procedures, we translated all the scales used in this study from English to Mandarin.

#### 2.2.1 Achievement goal orientation scale

In accordance with the needs of this study, the Task and Ego Orientation in Sport Questionnaire (TEOSQ) developed by Duda (1993) was selected. The AGO Scale includes Task Orientation and Ego Orientation. The scale, validated for reliability and validity, consists of 13 items, each rated on a five-point Likert scale. The scores for Task Orientation and Ego Orientation are summed separately; the higher the score, the greater the alignment with that orientation, thereby indicating an individual’s goal orientation tendency. The Cronbach’s *α* coefficient of this scale is 0.947, indicating good internal consistency reliability.

#### 2.2.2 Sports enthusiasm scale

The SE Scale used in this study was developed by Vallerand et al. (2003). The questionnaire includes 14 items, with 7 items measuring Harmonious Passion (HP) and 7 items measuring Obsessive Passion (OP), thus covering two dimensions. A seven-point Likert scale is used, ranging from 1 (“Not at all”) to 7 (“Very much”), with higher scores indicating greater levels of SE among students. The Cronbach’s α coefficient of the scale is 0.949, indicating good internal consistency reliability.

#### 2.2.3 Sports commitment scale

The SC Scale used in this study was adapted by Jung (1997) based on the ESCM (Expansion of the Sport Commitment Model) originally developed by Scanlan et al. (1993). The scale consists of 12 items, which include Cognitive Commitment (8 items) and Behavioral Commitment (4 items). A five-point Likert scale is used, where 1 to 5 represent increasing levels of agreement (1 = “Strongly Disagree” and 5 = “Strongly Agree”). The Cronbach’s *α* coefficient of this scale is 0.924, indicating good internal consistency reliability.

#### 2.2.4 Grit scale

The Original Grit Scale (Grit-O) developed by Duckworth et al. (2007) was used in this study. The questionnaire consists of 12 items, divided into two dimensions: Consistency of Interests and Perseverance of Effort, with each dimension comprising 6 items. The 6 items related to Consistency of Interests are reverse-scored. A five-point Likert scale is used for scoring: for positively worded items, responses range from 1 (“Not at all like me”) to 5 (“Very much like me”); for reverse-scored items, responses range from 1 (“Very much like me”) to 5 (“Not at all like me”). The Cronbach’s α coefficient for this scale is 0.942, indicating good internal consistency reliability.

#### 2.2.5 Perceived performance scale

The PP Scale was developed by Cho (2003) and consists of five items, forming a single-dimensional scale. A five-point Likert scale is used for scoring, with positively worded items scored from 1 (“Not at all like me”) to 5 (“Very much like me”). The Cronbach’s α coefficient for this scale is 0.882, indicating good internal consistency reliability.

#### 2.2.6 Control variables

In this study, gender, age, and educational level were selected as control variables. This is because they have been identified as potential factors influencing AGO, sport enthusiasm, sport commitment, perseverance and PP. These variables are often used in sport psychology research to reduce confounding effects and thereby elucidate the relationship between independent variables (e.g., AGO) and dependent variables (e.g., PP). Stults-Kolehmainen et al. (2013) found that gender and age significantly affect sport motivation and participation; Li et al. (2024) showed that increased education levels resulted in increased sport commitment and adherence; Tenenbaum et al. (2011) further emphasized the importance of controlling these demographic variables, noting that failure to account for these factors could lead to confusion and misleading conclusions when analyzing sports psychology variables. Therefore, including gender, age, and educational level as control variables in statistical analyses helps to reduce confounding effects, ensuring the validity and reliability of the findings.

Furthermore, variables including competition level, training intensity, and coaching influence were excluded from the data collection in this study, given that they were considered to exert a less direct impact on the psychological constructs under examination (Larkin et al., 2023). Prior studies have demonstrated that psychological attributes, such as AGO and grit, serve as relatively consistent predictors of PP and achievement among young athletes across varying training durations and competitive settings (Lochbaum et al., 2016). Although coaching influences may enhance motivation or effort, they typically modulate PP indirectly via psychological mediators rather than functioning as a primary determinant (Hwang et al., 2023). Secondly, acquiring data on competition level (e.g., amateur versus elite), training intensity (e.g., weekly training volume or effort), and coaching influences (e.g., style or support) would necessitate comprehensive evaluations, including training logs, coach-athlete interaction scales, or longitudinal monitoring; assessing these variables would demand substantial data collection efforts exceeding the scope and resources of this cross-sectional study. Consequently, demographic controls with well-established correlations were prioritized in this investigation.

#### 2.2.7 Data analytic strategy

The model was tested using Mplus 8.3 (Muthén and Muthén, 2017) as a statistical analysis tool. This is because Mplus provides the user with a comprehensive statistical toolbox for analyzing a wide range of models and most complex models can be analyzed with just a few Mplus commands (Javadizadeh, 2020).This study first performed a validated factor analysis (CFA) using Mplus, with CFA goodness-of-fit indices generally being CFI and TLI > 0.90 (Sun, 2005), SRMR < 0.08, RMSEA < 0.06 (Hu and Bentler, 1999), and χ2/df < 3 (Byrne et al., 1989). Second, we assessed the reliability of the scale using Cronbach’s alpha and the discriminant validity of the scale using Fornell and Larcker's (1981) criteria. We conducted Harman’s one-way test for common method bias in the data collected (Podsakoff et al., 2012). Third, we conducted a mediation analysis as suggested by Zhao et al. (2010) and used bootstrap analyses to test for mediation effects. Bias-corrected confidence intervals are considered more effective than traditional mediation tests in accurately evaluating indirect effects (MacKinnon et al., 2004). This study utilized a bootstrap method with 5,000 resamples and 95% bias-corrected confidence intervals to examine the significance of indirect effects in mediation analysis. The interval cannot contain a null in order to hypothesize a significant indirect effect (Preacher and Hayes, 2008).

## 3 Results

### 3.1 Reliability and validity

The present study conducted confirmatory factor analyses of Achievement Goal Orientation, SE, SC, Grit, and PP, and the results showed a good fit of the five-factor measurement model: χ^2^ = 2543.063, df = 1,466, *p* < 0.001, χ^2^/df = 1.73, CFI = 0.933, TLI = 0.929, and RMSEA = 0.044.

The reliability analyses in this study are listed on the diagonal in Table 1. The lowest Cronbach’s alpha coefficient for all scales in this study was 0.882. Therefore, the reliability of the scale does not seem to be an issue. Furthermore, the convergent validity was satisfactory as the composite reliability (CR) for each construct ranged from 0.89 to 0.96 (CR > 0.70) and the average variance extracted (AVE) for each construct ranged from 0.56 to 0.66 (AVE > 0.50), which exceeded the suggested thresholds. Discriminant validity was satisfactory because the square root of AVE was greater than the square root of all individual correlations. In addition, the results of the Harman one-way test showed that the variance explained by the first rotated factor was 38.6 per cent, which is less than 40 percent. Therefore, the questionnaire does not suffer from serious common method bias (Podsakoff et al., 2012; Gao et al., 2020).

**Table 1:** Descriptive statistics, correlations, and reliabilities.

Factor	*M*	SD	1	2	3	4	5
Achievement goal orientation	3.67	0.67	(0.947)				
Sports enthusiasm	3.57	0.65	0.472**	(0.949)			
Sports commitment	3.70	0.63	0.609**	0.553**	(0.924)		
Grit	3.57	0.64	0.506**	0.476**	0.625**	(0.942)	
Perceived performance	3.41	0.81	0.496**	0.536**	0.601**	0.544**	(0.882)

*N* = 377. SD is the standard deviation. The constructs’ internal reliabilities (alpha coefficients) are given in parentheses on the diagonal. **p < 0.01.

### 3.2 Descriptive statistics and correlation analysis

#### 3.2.1 Descriptive statistics (mean and standard deviation) and Pearson’s correlation for the scale

The results of descriptive statistics and correlation analysis for each variable are presented in Table 1. The results of the study indicated a significant positive correlation between AGO, SE, SC and Grit and PP with correlation coefficients ranging from 0.625 to 0.472, which provided the basis for our hypothesis testing. The coefficients of these variables are listed in Table 1. AGO was significantly positively correlated with SE (*r* = 0.472, *p* < 0.01), and was significantly positively correlated with SC (*r* = 0.609, *p* < 0.01), Grit (*r* = 0.506, *p* < 0.01), and PP (*r* = 0.496, *p* < 0.01). The significant positive correlation between SE and SC (*r* = 0.553, *p* < 0.01) was also significantly positively correlated with Grit (*r* = 0.476, *p* < 0.01) and PP (*r* = 0.536, *p* < 0.01). SC and Grit (*r* = 0.625, *p* < 0.01) were significantly and positively correlated with PP (*r* = 0.601, *p* < 0.01). Finally, Grit was significantly positively correlated with PP (*r* = 0.544, *p* < 0.01).

### 3.3 Chain mediating effects of SE, SC, and grit

#### 3.3.1 Structural model for testing hypotheses

**Figure 2:**
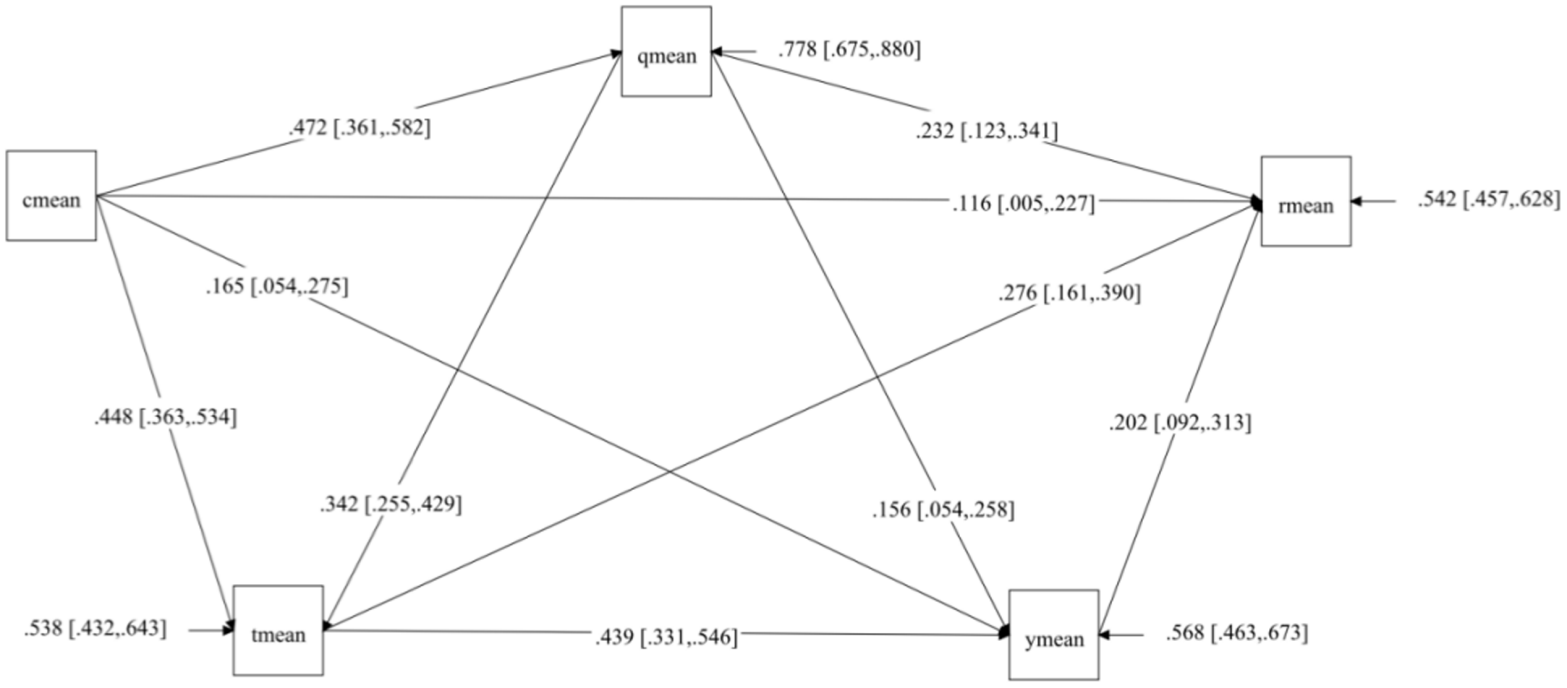
Structural equation model Diagr 1. cmean: achievement goal orientation; qmean: sports enthusiasm; tmean: sports commitment; ymean: grit; rmean: perceived performance.

Figure 2 presents the predicted values and significance of paths among the variables in the structural equation model: (1) AGO significantly predicted SE (*β* = 0.472, *p* < 0.001), SC (*β* = 0.448, *p* ≤ 0.001), Grit (*β* = 0.165, *p* < 0.001), and PP (*β* = 0.116, *p* < 0.001); (2) SE significantly predicted SC (*β* = 0.342, *p* < 0.001), Grit (*β* = 0.156, *p* < 0.001), and PP (*β* = 0.232, *p* < 0.001); (3) SC significantly predicted Grit (*β* = 0.439, *p* < 0.001) and PP (*β* = 0.276, *p* < 0.001); (4) Grit significantly predicted PP (*β* = 0.202, *p* < 0.001). These results indicate that the chain mediation path involving SE, SC, and Grit is valid.

To further examine potential mediating effects, the bias-corrected percentile Bootstrap method was used, with the number of iterations set to 5,000. According to the results, if the 95% confidence interval for the average path coefficient does not include zero, the effect is considered significant; if it includes zero, the effect is considered non-significant (Wen et al., 2004). Specific results can be found in Table 2. The direct effect value from AGO to PP was 0.116. The total mediating effect value was the sum of the mediating effects of the seven mediating paths, which amounted to 0.380. The total effect value was the sum of the direct effect and the total mediating effect, yielding 0.496 (Wen et al., 2004). The results of the mediation effect analysis indicate that both the direct and indirect effects from AGO to PP were significant. The proportion of the mediating effect in the total effect was 76.6%, meaning that 76.6% of the effect from AGO to PP occurred through the multiple mediation of SE, SC, and Grit. The proportions of effect values for the seven paths in the total effect were 25.0, 6.8, 22.0, 9.0, 8.0, 3.0, and 2.8%, respectively.

**Table 2:** Effect decomposition of the chain mediation model 2.

Path name	Indirect effect values	Bootstrap standard errors	*p*
Total effect	0.496	0.049	0.000
Direct effect	0.116	0.057	0.041
Indirect effect	0.380	0.048	0.000
Achievement goal orientation → Sports commitment → perceived performance	0.124	0.030	0.000
Achievement goal orientation → Grit → perceived performance	0.033	0.016	0.035
Achievement goal orientation → Sports enthusiasm → perceived performance	0.109	0.031	0.000
Achievement goal orientation → Sports enthusiasm → Sports commitment → perceived performance	0.044	0.012	0.000
Achievement goal orientation → Sports commitment → Grit → perceived performance	0.040	0.013	0.002
Achievement goal orientation → Sports enthusiasm → Grit → perceived performance	0.015	0.007	0.032
Achievement goal orientation → Sports enthusiasm → Sports commitment → Grit → perceived performance	0.014	0.005	0.007

## 4 Discussion

The methodology outlined in the Data Analytic Strategy section was employed in this study, utilizing Mplus 8.3 software for confirmatory factor analysis (CFA) and bootstrap analysis. CFA, combined with 5,000 bootstrap samples and 95% bias-corrected confidence intervals, ensured the model’s robustness and the accurate estimation of indirect effects. Harman’s single-factor test (38.6% < 40%) suggested no significant common method bias, whilst the composite reliability (CR > 0.70) and average variance extracted (AVE > 0.50) results confirmed the scale’s convergent and discriminant validity. The study’s findings (Table 2) demonstrated that AGO significantly influenced PP through SE, SC, and Grit (GRIT), supporting Hypothesis 1 (AGO positively influences PP), Hypotheses 2–4 (SE, SC, and Grit individually mediate the relationship), and Hypothesis 5 (chained mediation effects). Below, we discuss the theoretical and practical implications of these findings, the study’s limitations, and directions for future research.

### 4.1 Theoretical implications

Firstly, we investigated the influence of AGO on PP amongst swimmers and found that AGO factors exhibited a significant positive correlation with PP (Almagro et al., 2020). These findings are also evident in other sports. Comparative cross-motor analyses revealed significant context-dependence in the facilitation of PP by AGO (D’Astous et al., 2020). In team-based sports, such as football and basketball, social cognitive factors (e.g., teamwork efficacy) moderated the AGO–PP pathway (Alves et al., 2021). Adversarial programs, such as combat sports, are characterized by fluctuating AGO effects due to the dynamics of competitive pressures (Van Yperen, 2022). In contrast, the non-confrontational, aquatic environment of swimming minimizes direct competitive interference, rendering AGO more orientated towards enhancing PP through intrinsic drivers, such as skill improvement (Šilić et al., 2018). These variations indicate that, whilst the facilitation of PP by AGO is broadly applicable across sports, it remains more stable in swimming owing to its individualized nature.

Secondly, this study examined how AGO influences PP amongst swimmers through Sports SE. It was found that AGO significantly enhanced PP by fostering SE during training or competition (Chen and Ennis, 2009). This finding aligns with previous research across different sports. For instance, Jung et al. (2021), in their study of high school basketball players, observed that athletes’ emotional engagement significantly boosted PP when pursuing clear achievement goals. In swimming, a highly autonomous individual sport, AGO primarily manifests as a sustained pursuit of personal skill development, with SE being driven by an individual focus on the training process and a sense of accomplishment rather than social support derived from teamwork (Pluhar et al., 2019). In addition the cultural context influences the mechanisms by which AGO stimulates SE (Almagro et al., 2015). For example, in East Asian collectivist cultures, such as Japan and China, where enthusiasm for sport partly depends on social recognition, cultural tendencies may influence how AGO manifests across different settings (Lau et al., 2007; Ji et al., 2024). Compared to other individual sports, for example, SE in tennis stems mainly from the sense of achievement when using strategies during a match (Hammes and Link, 2024). The uniqueness of swimming lies in its repetitive training in the water, and AGO fuels SE by pushing athletes to focus on improving their technique and feeling the progress, which further enhances their confidence and positive evaluation of their performance (Soulliard et al., 2019; McNarry et al., 2021). Thus, SE mediates the effect of AGO on PP in swimming, with its expression shaped by sport type, cultural background, and the unique training environment.

Thirdly, this study explored the positive effects of AGO on swimmers’ PP through SC, which was found to play a partially mediating role. Consistent with prior research, AGO enhances SC in swimming training through task orientation and goal pursuit (Biddle et al., 2003), which subsequently boosts PP by strengthening athletes’ confidence in their competitive abilities (Kong et al., 2022). In contrast, SC in basketball stems more from teamwork support, which enhances athletes’ PP. The results of this paper have also been validated in the field of education, e.g., students focused on mastering the learning content (e.g., understanding complex topics), and by enjoying the learning process, they increased their confidence in their abilities (Pekrun et al., 2017). Research on flow theory suggests that athletes are more likely to enter a flow state when challenges align with their skill levels, thereby fostering greater SC and elevating PP (Jackson and Csikszentmihalyi, 1999). This indicates that providing appropriate challenges that align with an individual’s skill level helps promote SC and enhance PP. Therefore, increasing the level of SC can be considered a strategy for enhancing athletes’ competitive level. Through training that increases SC, an improvement in competitive performance can be expected.

Fourthly, this study investigated how AGO amongst swimmers positively influences PP through Grit. This finding is supported by existing literature: by exploring motivational mechanisms in athletic contexts, AGO fosters sustained effort and heightened motivation in students, enabling them to perceive training setbacks as opportunities for growth and promoting long-term persistence (Harnar et al., 2021). This finding provides a basis for the idea that AGO promotes sustained effort in this study. AGO enhances athletes’ determination to pursue their goals despite challenges, aligning with the pathway through which AGO affects PP via Grit in this research (Martínez-González et al., 2021). Recent studies have further reinforced this outcome, demonstrating that AGO reduces anxiety and enhances PP by elevating Grit levels during competitive exercise, a mechanism consistent with perseverance acting as a mediator in the present study (Apró et al., 2024). Similarly, these findings are corroborated in workplace settings; for instance, employees who maintain focus and tenacity whilst tackling complex tasks significantly enhance their self-assessed performance ratings (Jordan et al., 2019). This result is consistent with the role of fortitude in this study. Thus, the mechanism by which Grit mediates AGO and PP not only holds true in the field of swimming, but also reflects cross-domain motivational and behavioral patterns, providing important theoretical insights for multidisciplinary research.

Fifthly, this study revealed that SE and SC serve as mediators in the relationship between AGO and PP. AGO fosters SE by enhancing the sense of meaning derived from the activity (St-Cyr et al., 2021). Previous research has shown that individuals with AGO focus on skill acquisition, resulting in stronger interest in the activity, this interest usually manifests itself in a passionate commitment to sport (Chin et al., 2012). Furthermore, SE was found to be significantly and positively correlated with SC (Uğraş et al., 2024). Specifically, athletes with enthusiasm were more inclined to view sport as part of their self-identity and thus demonstrated a higher willingness to participate consistently. According to the SC Model, highly committed athletes tend to invest more time and effort, and this persistence bolsters their confidence in their abilities, ultimately enhancing PP (Scanlan et al., 1993). Notably, these mediating effects may vary by sport type: team sports (e.g., soccer) might amplify SC through social bonds (Zuckerman et al., 2021), while individual sports (e.g., tennis) may emphasize SE driven by intrinsic motivation (Šagát et al., 2021). Thus, AGO indirectly influences PP through the interlocking mediating effects of SE and Sport Commitment.

Sixthly, this study demonstrates that SE and Grit mediate the relationship between AGO and PP. Reviewing previous literature, a significant positive correlation between SE and grit was found in a study of 71 footballers, suggesting that enthusiastic athletes tended to show greater perseverance, which is consistent with our findings (Loftesnes et al., 2021). Athletes passionate about physical activity display heightened interest and engagement, which bolsters their Grit in pursuing their goals (Uğraş et al., 2024). Athletes with Grit consistently invest effort, enhancing their competitiveness and confidence in their abilities, thereby improving PP (Lochbaum et al., 2022). While previous research supports the use of SE and Grit as mediators (Chin et al., 2012; Guo et al., 2024), our chain of mediators from AGO to SE and Grit to PP provides a novel integrative model that has been less elaborated upon in previous research. The results of this study shed light on the chain mechanism by which AGO indirectly enhances PP by stimulating SE and further enhancing Grit.

Seventhly, this study established that SC and Grit mediate the relationship between AGO and PP. Athletes’ cognitive models of success in competitive contexts and their behavioral tendencies foster sustained commitment to training and competition, enhance adherence to long-term goals, and consequently improve PP. A meta-analysis demonstrated that athletes with AGO exhibit greater effort and perseverance in pursuing their goals, resulting in enhanced athletic performance, a finding consistent with the present research (Puente-Díaz, 2012). Additionally, previous research in the literature has shown that sport commitment significantly enhances grit in young athletes, further supporting this mediating mechanism (Konter and Ng, 2012). Research in education suggests that academic commitment markedly enhances students’ academic performance by fostering resilience, a process mirrored in physical education settings (Xu et al., 2023). Similarly, organizational behavior research reveals that organizational commitment strengthens employees’ Grit, which in turn improves performance, pointing to a shared psychological foundation for the mediating effects observed in this study (Jung et al., 2020). Thus, the present study reveals the psychological pathways by which AGO enhance PP through SC and Grit.

Eighthly, this study confirmed the multiple chain mediating roles of SE, SC, and Grit between AGO and PP. Achievement Goal Theory (AGT) posits that AGO shapes an individual’s performance by guiding their goal-directed behavior (Elliot and Hulleman, 2017). Specifically, AGO enhanced swimmers’ enthusiasm for the sport, In particular, mastery goal orientated swimmers showed higher training participation due to intrinsic interest (Hwang et al., 2019). Enthusiasm for sport, in turn, enhances commitment to sport, a process that is consistent with the mechanism in AGT where mastery goals drive sustained effort (Roberts et al., 2018). SC fosters Grit by promoting training consistency, which manifests as persistent pursuit of long-term goals (Tedesqui and Young, 2017). Grit ultimately enhances PP, as its sustained effort bolsters swimmers’ positive self-evaluations of performance, particularly in endurance-based events (Roshini and Zina, 2019). Thus, AGO promotes PP through SE, SC, and Grit.

### 4.2 Practical implications

This study explored the pathways of SE, SC and Grit in the relationship between AGO and PP, and provided a practical guide for coaches to enhance PP in college athletes. The following step-by-step coaching strategies, grounded in the principles of AGO (which emphasizes goal-directed effort and achievement), enhance SE, SC, and Grit within a realistic sporting context: Step 1: Establishing Challenging Goals to Boost SE. Coaches should leverage AGO to set clear, challenging short-term goals that motivate athletes. For instance, during a 10-week swimming program, coaches might establish weekly targets (e.g., reducing the 100-metre freestyle time by 2 s) and monitor progress via timed assessments, fostering SE through visible improvement. Step 2: Emphasize Personal Progress to Enhance Sport Commitment. Encourage athletes to focus on personal progress rather than competition results through AGO, using regular assessments to enhance sport commitment. For example, monthly technical (e.g., stroke consistency) and psychological (e.g., self-confidence) progress checks highlight personal growth to reinforce training commitment. Step 3: Provide Specific Feedback to Maintain Sport Enthusiasm and Sport Commitment. Providing detailed, actionable feedback based on AGO sustains SE and Sport Commitment in athletes. For example, after a practice session, a coach could say ‘you improved your turn speed by 0.5 s today, next time focus on a faster stomp on the wall’, associating effort with progress to maintain enthusiasm and commitment. Step 4: Fostering a Supportive Team Environment to Enhance Grit. Grit is strengthened through collective support within AGO-driven team interventions. For example, a season-long paired training initiative, where swimmers collaborate to achieve a shared endurance goal (e.g., completing 20 laps together), cultivates perseverance and resilience under pressure. These achievement-based goal-orientated strategies provide coaches with a structured approach to ultimately enhance athletes’ motivation and PP by increasing SE, SC and Grit.

### 4.3 Limitations and future recommendations

This study has several limitations. Firstly, this study only collected samples from Tianjin and Beijing, and the generalizability of the findings may be limited. Therefore, future research should expand the sample to include athletes from different cultures and regions to validate the applicability of these psychological variables in a broader context. Secondly, while focusing on key mediators, we overlooked confounding variables such as anxiety, which may intensify PP pressure, or coaching support, which could strengthen goal orientation effects. Additionally, gender, age, competitive experience, and training level likely moderate the AGO–PP relationship. For instance, gender may influence motivational tendencies (e.g., males prioritizing task goals, females ego goals) (Clemente-Suárez et al., 2021), age may affect goal-setting adaptability (e.g., younger athletes adjusting more readily) (Ji et al., 2024), competitive experience may improve goal-to-performance translation (e.g., through honed skills) (Levental et al., 2023), and training level may sustain effort (e.g., advanced athletes enduring longer) (Morici et al., 2016). These confounding and moderating effects warrant deeper exploration in future studies to clarify their roles. Thirdly, dependence on self-reported data introduces potential biases that could undermine the accuracy of the findings. Participants’ responses to constructs such as Grit and PP are susceptible to social desirability bias, whereby individuals may exaggerate positive attributes (e.g., Grit or PP) to align with perceived expectations, potentially amplifying their associations (Meisters et al., 2020). Likewise, recall bias may skew reports of SE or SC owing to inaccurate recollection of past experiences, while the subjective nature of self-assessment could engender variability in participants’ interpretation and evaluation of their performance, thereby diminishing measurement consistency. Future studies could address these biases by incorporating objective measures, including coach assessments, behavioral observations, or physiological markers (e.g., heart rate variability as an indicator of effort), to corroborate self-reported data. Lastly, the cross-sectional design imposes constraints that limit the scope of this investigation. Although this cross-sectional study of swimmers substantiates the hypothesized relationships among AGO, SE, SC, Grit, and PP, its design precludes the establishment of causality among these variables. Moreover, single time-point data collection fails to capture temporal dynamics, such as Grit’s potential growth over a season or SE’s variation across training phases, and may be confounded by transient factors (e.g., recent competition outcomes), potentially skewing results. Future research should adopt longitudinal designs, tracking these variables across multiple time points (e.g., pre-season, mid-season, and post-season), or experimental methods manipulating AGO to assess its impact on SE and PP, thereby addressing these limitations.

## 5 Conclusion

This study establishes that AGO significantly enhances PP among swimmers, with SE, SC, and Grit serving as interconnected mediators in a chain multiple mediation process. These findings confirm the proposed relationships, demonstrating that AGO fosters athletic improvement through the combined influence of SE, SC, and Grit. The interrelated roles of these factors provide a basis for the development of targeted psychological strategies to enhance swimmers’ PP and contribute to a deeper understanding of the dynamics of swimming achievement goals.

## Data Availability

The raw data supporting the conclusions of this article will be made available by the authors, without undue reservation.
